# Editorial: Heart failure induced by non-cardiac therapies

**DOI:** 10.3389/fphar.2026.1943353

**Published:** 2026-07-29

**Authors:** Mohammed Mashali, Can Yerebakan, Anthony Zulli, Nancy Saad

**Affiliations:** 1 Center for Cardiovascular Research, The Abigail Wexner Research Institute, Nationwide Children’s Hospital, Columbus, OH, United States; 2 The Heart Center, Nationwide Children’s Hospital, Columbus, OH, United States; 3 Department of Surgery, Faculty of Veterinary Medicine, Damanhour University, Damanhour, Egypt; 4 Department of Cardiothoracic Surgery, Nationwide Children’s Hospital, Columbus, OH, United States; 5 School of Health Sciences, The University of Notre Dame Australia, Sydney, NSW, Australia; 6 Institute for Health and Sport (IHES), Victoria University, Melbourne, VIC, Australia; 7 Department of Physiology and Cell Biology, College of Medicine, The Ohio State University, Columbus, OH, United States; 8 Department of Pharmacology and Toxicology, Faculty of Pharmacy, Capital University, Cairo, Egypt

**Keywords:** biomarker surveillance, cardio-oncology, cardiotoxicity, cardiovascular risk, heart failure, hemodialysis, inflammation, non-cardiac therapies

Heart failure induced or aggravated by therapies used for non-cardiac diseases is an increasingly important challenge in modern clinical practice. As treatments for cancer, renal failure, metabolic disease, inflammatory disorders, and other chronic conditions continue to improve survival and long-term disease control, cardiovascular complications may emerge during or after otherwise necessary therapies. These complications may occur through direct myocardial toxicity, hemodynamic stress, systemic inflammation, neurohormonal activation, metabolic disturbance, endothelial dysfunction, or interactions between treatment exposure and pre-existing cardiovascular risk factors. This Research Topic, ‘Heart Failure Induced by Non-Cardiac Therapies,’ brings together a collection of articles that address this problem from clinical, therapeutic, and mechanistic perspectives. Current heart failure and cardio-oncology guidelines increasingly emphasize prevention, baseline cardiovascular risk assessment, early recognition of treatment-related cardiac dysfunction, and coordinated longitudinal care (Heidenreich et al., 2022; Lyon et al., 2022).

A major theme of this Research Topic is the need for early cardiovascular surveillance in patients exposed to potentially cardiotoxic therapies. In the cardio-oncology setting, Fazzini et al. examined soluble suppression of tumorigenicity-2 (sST2) changes during cardiotoxic cancer treatment in a systematic review and meta-analysis. Their work evaluated patients treated with anthracyclines and/or human epidermal growth factor receptor 2 (HER2)-directed therapies and compared longitudinal sST2 patterns with more established biomarkers, including troponin and N-terminal pro-B-type natriuretic peptide (NT-proBNP). Although troponin showed greater early longitudinal variation, sST2 showed time-dependent variation and was inversely correlated with left ventricular ejection fraction. These exploratory findings support the potential role of sST2 as an adjunct biomarker within multimarker surveillance strategies, while also highlighting the need for standardized longitudinal studies in patients who do and do not develop cardiotoxicity. These findings are consistent with contemporary cardio-oncology guidance emphasizing longitudinal surveillance using clinical evaluation, cardiac imaging, and biomarkers before overt heart failure develops (Lyon et al., 2020; Lyon et al., 2022).

The Research Topic also highlights heart failure risk in patients receiving life-sustaining non-cardiac therapies. Hou and Chen investigated risk factors and risk-management strategies in maintenance hemodialysis patients complicated by heart failure. Their study identified age, hyperglycemia, inflammation, and comorbidities such as hypertension, diabetes, and coronary heart disease as important contributors to heart failure risk, with age, glycated hemoglobin (HbA1c), and C-reactive protein emerging as independent predictors in multivariate analysis. Importantly, the authors also assessed a targeted risk-management approach and reported improvements in psychological status, complications, quality of life, and nursing satisfaction. These findings support the value of risk-oriented care models that combine clinical, biochemical, and patient-centered outcomes in high-risk treatment settings. This article emphasizes that treatment-associated heart failure is not limited to direct drug toxicity; it may also occur or worsen in settings where chronic therapy imposes repeated hemodynamic and metabolic stress on vulnerable patients, consistent with the broader recognition that chronic kidney disease and kidney replacement therapy require integrated cardiovascular risk reduction and multidisciplinary care (Stevens et al., 2024).

Two articles in this Research Topic focus on adjunctive therapeutic strategies for patients with established heart failure. Deng et al. conducted a network meta-analysis of randomized controlled trials evaluating plant extracts in heart failure. Their findings suggested that different extracts may influence distinct outcomes, including 6-min walk distance, B-type natriuretic peptide, quality of life, left ventricular ejection fraction, New York Heart Association functional class, and inflammatory markers. Huang et al. evaluated *Salvia miltiorrhiza* and ligustrazine injection as an adjunct to conventional therapy in a systematic review and meta-analysis. Their analysis reported improvements in cardiac function, natriuretic peptide levels, inflammatory markers, endothelial function, and exercise capacity, as well as fewer cardiovascular adverse events, without a clear increase in overall adverse reactions. Together, these studies suggest that adjunctive therapies may have potential in selected patients with established heart failure, particularly when they target inflammation, endothelial dysfunction, oxidative stress, and cardiac remodeling. However, both articles also underscore important evidence limitations, including heterogeneity, methodological concerns, and the need for larger, higher-quality randomized trials with standardized protocols and long-term follow-up before broad clinical adoption.

Mechanistic understanding remains essential for improving prevention and treatment. Swiderski et al. reviewed the alpha-7 nicotinic acetylcholine receptor (α7nAChR) as a potential therapeutic target in heart failure, with particular attention to heart failure with preserved ejection fraction. The review connects α7nAChR signaling with the cholinergic anti-inflammatory pathway, renin–angiotensin–aldosterone system activation, neurocardiac signaling, and metabolic dysfunction. These pathways are highly relevant to therapy-associated heart failure because many non-cardiac treatments and chronic disease states can affect the heart through systemic inflammation, autonomic imbalance, vascular injury, and maladaptive remodeling. This article therefore provides a useful mechanistic framework for future therapeutic development by highlighting neuroimmune and cardiometabolic pathways that extend beyond conventional hemodynamic targets.

Collectively, the articles in this Research Topic show that heart failure induced, aggravated, or clinically shaped by non-cardiac therapies is a multidimensional clinical problem. The contributing studies span cancer therapy-related cardiotoxicity, hemodialysis-associated risk, adjunctive treatment approaches, and neuroimmune mechanisms with therapeutic relevance. Across these areas, several shared priorities are evident: identifying high-risk patients before clinical deterioration, improving longitudinal monitoring, integrating biomarkers with clinical and imaging data, and developing interventions that address inflammation, endothelial dysfunction, neurohormonal activation, and remodeling.

This Research Topic also emphasizes the importance of multidisciplinary care. Preventing and managing treatment-associated heart failure requires coordination among cardiologists, oncologists, nephrologists, pharmacologists, nurses, primary care clinicians, and other healthcare professionals (Heidenreich et al., 2022; Lyon et al., 2022). As therapies for non-cardiac diseases continue to expand, cardiovascular safety should remain an integral part of treatment planning and follow-up. Future research should prioritize prospective designs, standardized definitions of treatment-related cardiac dysfunction, longer follow-up, and integration of clinical, biomarker, imaging, and mechanistic data.

In conclusion, this Research Topic advances the discussion of heart failure induced, aggravated, or clinically shaped by non-cardiac therapies by linking risk identification, biomarker surveillance, therapeutic mitigation, and mechanistic discovery. The articles collectively support a shift toward proactive cardiovascular protection in complex patients receiving non-cardiac therapies. Continued research in this area will be essential to improve early detection, personalize risk reduction, and preserve both survival and quality of life.
